# Ranibizumab after laser photocoagulation failure in retinopathy of prematurity (ROP) treatment

**DOI:** 10.1038/s41598-017-12264-z

**Published:** 2017-09-19

**Authors:** Anna Gotz-Więckowska, Anna Chmielarz-Czarnocińska, Marta Pawlak, Janusz Gadzinowski, Jan Mazela

**Affiliations:** 1 Department of Ophthalmology, Poznań University of Medical Sciences, Poznań, Poland; 2 Head of Chair and Department of Neonatology, Poznań University of Medical Sciences, Poznań, Poland; 3 Department of Neonatology and Infectious Diseases, Laboratory of Microbiome and Nutrition, Poznań University of Medical Sciences, Poznań, Poland

## Abstract

The purpose of this study was to investigate the anatomical and functional outcomes of the two-stage treatment of severe retinopathy of prematurity (ROP) using laser photocoagulation and intravitreal ranibizumab injection. The medical records of 53 eyes of 28 infants treated by conventional laser photocoagulation with deferred intravitreal 0.25 mg/0.025 mL ranibizumab injection were analysed. All patients had at least 11 months of follow-up. In the analysed group, the mean gestational age at birth was 25 weeks and mean birthweight was 790 g. The mean time of laser photocoagulation was 34 weeks of postmenstrual age (PMA). Ranibizumab injection was performed on average at 37 weeks of PMA. The mean time between interventions was 19 days. Retinal detachment occurred in 12 eyes (22.6%), in three children bilaterally. Visual responses were obtained in 23 of 28 treated children. Our results indicate that ranibizumab injection can be taken into consideration in the selected cases of laser photocoagulation failure. The unsatisfactory results of this study elicited a change in the ROP treatment protocol in our medical centre. The study gives an insight into anatomical and functional outcomes of ROP treatment in the Central and Eastern European population.

## Introduction

Despite significant progress in the treatment methods, retinopathy of prematurity (ROP) is a disease that still causes blindness or impaired vision in children. Gestational age and the birth weight of the patient suffering from ROP vary considerably depending on the country of origin^1^. There is little epidemiological data summarising the results of ROP treatment in Central and Eastern Europe.

Good functional and anatomic results depend on the implementation of the treatment at the appropriate moment, but in the case of aggressive forms of ROP located in zone I, even timely photocoagulation is not always successful. According to the newest scientific reports better results can be obtained using bevacizumab monotherapy compared to laser ablation^2^; however, this treatment remains controversial^3^. A much-debated question is whether and to what extent the anti-vascular endothelial growth factor (anti-VEGF) therapy affects the further development of children. Morin *et al*.^4^ examined patients at 18 months corrected age and concluded that those treated with intravitreal injections had higher odds of severe neurodevelopmental disabilities compared to these who underwent laser photocoagulation.

The increasing number of aggressive posterior ROP (APROP) and zone I ROP is related to the improvement of neonatal care and the increased survival of ultrapremature infants (gestational age <26 weeks, birth weight <750 g, or both)^5^. In such cases, complete laser ablation may not be possible in a single session, and repeat laser ablation may be warranted. The percentage of treatment failure in this group of patients is high and reaches 45%^6,7^. Therefore, there is an incentive for the search for new treatment protocols which would improve the prognosis in this group of patients.

In the search for the optimal treatment, ophthalmologists have attempted to use both anti-VEGF therapy and laser photocoagulation. The treatment possibilities include laser ablation followed by a second course of laser treatment^8^, injections of anti-VEGF without laser ablation^2,9^, a combination of these therapies applied simultaneously^10^, combined therapies in which laser ablation is complemented by anti-VEGF injection in the case of laser failure^11,12^, and combined therapies in which laser ablation is used in the case of injection failure^13^.

Until May 2011 laser ablation was the only therapeutic procedure available in Poznań University of Medical Sciences medical centre. We introduced anti-VEGF injections in the most challenging cases as a salvage, experimental treatment to inhibit progressive ROP in the eyes with no or transient improvement after laser photocoagulation. The availability of anti-VEGF agents was at that time limited. The aim of this study was to analyse the anatomical and functional outcomes of this two-stage ROP treatment protocol in our centre.

## Methods

The study followed the tenets of the Declaration of Helsinki and was approved by the Bioethics Committee of the Medical University in Poznań (Resolution No. 535/10).

This was a single centre, retrospective study conducted at Neonatal Intensive Care Unit (NICU) at the Poznań University of Medical Sciences, Poland. The medical records of 1248 patients screened for ROP between May 2011 and August 2015 were evaluated. In this period, premature infants with ROP progression despite laser treatment (with persistent plus disease and failure of regression of retinal neovascularisation) or patients in whom photocoagulation was performed too late (children transferred from other medical centres) had intravitreal ranibizumab administered as an adjuvant therapy.

The screening examination followed the guidelines of the National Specialist Team in Paediatrics and School Medicine and Polish Ophthalmological Society for ROP screening (≤33 weeks of postmenstrual age, ≤1800 g of birth weight or on the request of neonatology specialist due to high risk of ROP)^14^. The first examination was undertaken at 4 weeks of chronological age, with further examinations depending on the condition of the eyes every 7–10 days.

The treatment criteria were based on the Early Treatment for Retinopathy of Prematurity (ETROP) guidelines^15^. Treatment was applied in the eyes with severe – type I according to ETROP – forms of ROP: any stage of ROP in zone I with plus disease; zone I, stage 3 ROP without plus disease; or zone II, stage 2 or 3 ROP with plus disease. Aggressive posterior ROP (APROP) also fulfilled the criteria for the treatment. It has been defined by the International Classification of Retinopathy of Prematurity (ICROP) as an aggressive form of ROP seen predominantly in zone I or posterior zone II, which presents with features that include plus disease in all four quadrants, posterior disease out of proportion to the peripheral retinopathy and rapidly progressive vascular changes along with flat neovascularisation (FNV), intraretinal shunting without ridge tissue, haemorrhages, and the tendency to progress to retinal detachment without evolving through the typical stages 1 to 3^8^.

In the first stage of treatment, retinal photocoagulation with a diode laser of a wavelength of 810 nm with confluent burns (IRIS Medical Instruments Inc.) was performed on the avascular retina. If the laser ablation failed the second line of treatment – ranibizumab injection – was applied. After sterilisation with 5% povidone/iodine solution and insertion of a lid speculum, a dose of 0.25 mg/0.025 mL of ranibizumab was injected into the infero-temporal quadrant using a 30 gauge needle. The off-label use of anti-VEGF and the potential risks were discussed with the parents and their written informed consent was obtained. Both procedures were performed under general anaesthesia. After the injection, and every 6 hours for the next 5 days, topical moxifloxacin was administered. The infants were examined 1 day after the procedure and every 7–10 days until total regression of ROP.

The ophthalmological examinations were first performed during the stay of the patients at NICU and after discharge from the hospital in the Outpatient Clinic for Preterm Infants of University Clinical Hospital no. 1 in Poznań. All treated infants had a continuous follow-up with the treating team. Visual reactions were evaluated individually depending on the age of a child, also taking into account existing disabilities.

The datasets generated during and/or analysed during the current study are available from the corresponding author on reasonable request.

### Statistical analysis

A statistical analysis of the factors that may correlate with the development of retinal detachment in ROP was performed. The following clinical features were studied: gestational age (GA; weeks), birth weight (BW, grams); occurrence of APROP, retinopathy of prematurity in zone I, occurrence of bronchopulmonary dysplasia (BPD), intraventricular hemorrhage (IVH), necrotizing enterocolitis (NEC), patent ductus arteriosus ligation (PDA ligation), periventricular leukomalacia (PVL). The results are presented as percentages of categorical variables or a median (range) for continuous variables using Kolmogorov–Smirnov test. A P value of less than 0.05 was considered significant. The chi-square test was used to evaluate association between retinal detachment and categorical variables. Statistical analysis was performed using Statistica version 10, 2011 (Stat Soft, Inc., Tulsa, Oklahoma, United States).

## Results

### Clinical characteristics of the patients

**Figure 1:**
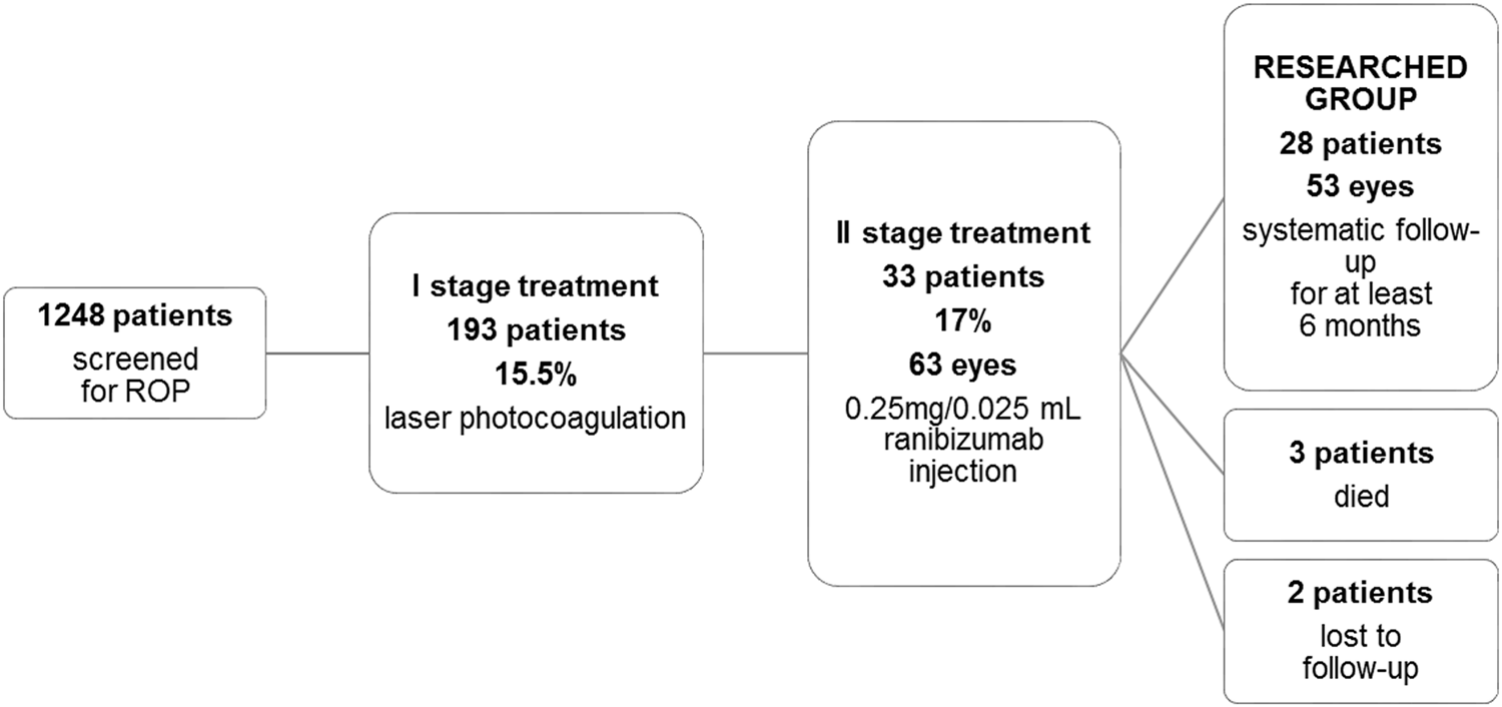
Patient flow diagram.

Among 1248 infants screened for ROP, 193 patients (15.5%, 386 eyes) were qualified for laser photocoagulation of the retina. In 160 patients (83%, 320 eyes) treated with laser therapy, total regression of ROP was observed. In 2 children, laser ablation was applied twice. In 33 patients (17%, 63 eyes), ranibizumab injection was used as the second line of treatment. Of them, 3 patients died from complications from systemic conditions and two were lost to follow-up. Finally, the study group included 28 children (53 eyes) who underwent laser ablation and, as a second step intervention, ranibizumab injection and had at least 11 months of follow-up. Three children had ranibizumab injection administered in one eye. The patients were regularly examined in the outpatient clinic. The flow chart of the study population is shown in Fig. 1.

The study group consisted of 8 girls and 20 boys, 25 from a singlet pregnancy and three from multiple pregnancies (one twin, two triplets). The mean gestational age (GA) at birth of studied infants was 25 ± 2 weeks (23 to 30 weeks), the mean birth weight (BW) was 790 ± 243 g (480 to 1450 g). The mean time at which laser photocoagulation was performed was 34 ± 1 weeks of postmenstrual age (32–38 weeks), at 67 ± 11 days of life (46–88 days). After laser ablation failure, ranibizumab injection was performed, on average at 37 ± 2 weeks PMA (33–43 weeks), at 86 ± 16 days of life (57–135 days). The mean time between interventions was 19 ± 10 days (2–48 days). There were no systemic or local complications of the treatment. The clinical characteristics of 28 infants included in the study are shown in Table 1.

**Table 1:** The clinical characteristics of 28 infants included in the study.

Case/Sex	Int/Ext	GA, weeks	BW, g	Stage/Zone	Plus/Pre-plus	Age at Laser, days	PMA at Laser, weeks	Ranibizumab Injection, eye	Age at Ranibizumab Injection, days	P MA at Ranibizumab Injection, weeks	Time Span between Procedures, days	Retinal Detachment	Visual Function	Follow-up, months
1/F	int	23	480	APROP/I	plus	74	33	+/+	81	34	7	+/+	−	31
2/F	ext	23	610	APROP/I	plus	80	34	+/+	86	35	6	−/−	+	24
3/M	int	23	665	APROP/I	plus	70	33	+/+	77	34	7	−/−	+	38
4/M	int	24	540	APROP/I	plus	87	36	+/+	108	39	21	−/−	+	15
5/F	int	24	620	APROP/I	plus	87	36	−/+	135	43	48	−/	−	11
6/M	int	24	640	3/I	plus	69/88	33/36	+/+	97	37	28	+/+	−	57
7/M	int	24	685	3/cII	plus	80	35	+/+	104	38	24	+/−	+	66
8/M	int	24	750	3/cII	plus	65	33	+/+	86	36	21	−/−	+	36
9/M	int	24	760	3/I	pre-plus	64	33	+/+	94	37	30	−/−	+	25
10/M	int	24	780	1/I	pre-plus	64	33	+/+	73	34	9	−/−	+	13
11/M	int	24	887	3/II	plus	59	32	+/+	83	35	24	−/−	+	39
12/F	int	25	550	3/I	plus	65	34	+/+	91	38	26	−/+	+	65
13/M	int	25	585	3/II	plus	72	35	+/+	94	38	22	−/−	+	24
14/M	int	25	767	1/I	plus	75	35	+/+	82	36	7	+/−	+	14
15/M	int	25	770	APROP/I	pre-plus	64	34	−/+	92	38	28	/+	−	53
16/F	int	25	785	3/cII	plus	50	32	+/+	57	33	7	−/−	+	32
17/M	int	25	810	1/I	plus	71	35	+/+	104	39	33	−/−	+	29
18/F	int	25	825	1/I	plus	58	33	+/+	72	35	14	−/−	+	21
19/M	int	25	830	2/cII	plus	57	33	+/+	78	36	21	−/−	+	25
20/F	ext	25	850	APROP/I	plus	67	34	+/+	76	35	9	+/−	+	55
21/M	ext	25	870	3/II	plus	71	35	+/+	83	36	12	−/−	+	36
22/M	int	26	520	3/I	pre-plus	73	36	+/+	95	39	22	−/−	+	13
23/M	int	26	620	3/I	plus	66	35	+/−	93	39	27	−/	+	39
24/F	int	26	750	3/I	plus	57	34	+/+	71	36	14	−/+	+	38
25/M	int	26	1105	3/II	plus	66	35	+/+	87	38	21	−/−	+	16
26/M	int	27	1225	2/I	pre-plus	46	33	+/+	74	37	28	−/−	+	16
27/M	ext	28	1400	3/I	plus	52	35	+/+	66	37	14	+/+	−	65
28/M	ext	30	1450	3/II	plus	61	38	+/+	63	39	2	−/−	+	63

Abbreviations: F, female; M, male; Int, internal; Ext, external; GA, gestational age; BW, birth weight*; cII, central II zone;* PMA, postmenstrual age; Ranib. Inj., ranibizumab injection.

Visual function: “+” meaning at least light perception or more advanced reaction; “−” meaning no response to visual stimuli.

The enrolled children had a systematic follow-up which ranged from 11 to 66 months. The corrected age of the patients at the moment of the enrolment to the study was between 7.5 months and 5 years.

Of the 28 patients, 23 were born at the Gynaecology and Obstetrics Hospital of Poznań University of Medical Sciences or were transferred from other medical centres within the first hours of life. Five children were transferred after the diagnosis of ROP requiring treatment was made. Laser photocoagulation was applied the day after their admission to our medical centre. Two such patients had high vascular proliferations within 12 clock hours area of the central zone II and plus disease (iris vascular engorgement, poor pupillary dilatation, vascular dilatation and tortuosity were present in 4 quadrants of the eye) at the time of admission for the treatment. In one of them, ranibizumab injection was applied 14 days after laser ablation (it was the first patient in whom we used ranibizumab and the long interval between procedures was due to organisational difficulties). The second patient received ranibizumab within two days after laser treatment. In the first child, retinal detachment occurred bilaterally and reattachment was not obtained after vitrectomy. In the second child, the retina remained attached but bilateral macular ectopy occurred. The remaining 26 patients had treatment-requiring forms of ROP located mostly in zone I and plus disease.

### The course of ROP

In 22 children, 43 eyes (in one child ranibizumab was injected in one eye), there was no or little improvement in vessel condition after laser therapy. In this group, the average time between laser therapy and ranibizumab injection was 15 ± 8 days (2–28 days). ROP reoccurred - after a period of transient remission they again met the criteria for treatment listed by ETROP guidelines - in 6 children, in 10 eyes (in two children ranibizumab was injected in one eye). In this group, the average time between laser therapy and a ranibizumab injection was 31 ± 9 days (from 22–48 days). We did not observe proliferative membranes in these children.

### Retinal detachment

Retinal detachment was observed in 9 children, i.e. in 12 (22.6%) of 53 eyes treated with laser photocoagulation and ranibizumab injection, in 3.1% of all treated eyes, and in 3 children bilaterally. Further treatment (vitrectomy) was undertaken in 5 out of 12 eyes (42%). Three children (5 eyes) had APROP, 4 children (4 eyes) had ROP in zone I with plus disease, 1 child (1 eye) had stage 3 ROP in central zone II and one child (2 eyes) was transferred from another centre with advanced vascular proliferation in zone I and plus disease. This child differed significantly as far as gestational age and birth weight compared to the remaining patients in the study group (GA 28 weeks, BW 1400 g). The data on profiles of patients with and without retinal detachment according to IC-ROP (international classification of ROP) are shown in Table 2, all children enrolled to this study had either plus or pre-plus disease. The comparison of the clinical features of the infants with and without retinal detachment indicated that the two groups differed significantly only as far as visual response rate was considered being lower in the retinal detachment group. No other clinical features correlated statistically significantly with retinal detachment. The clinical characteristics of the patients with and without retinal detachment are shown in Table 3.

**Table 2:** Profiles of patients with and without retinal detachment.

Zone	Stage	Patients with retinal detachment (N = 9) N, (%)	Patients without retinal detachment (N = 19) N, (%)
I	APROP	3 (33.3)	4 (21.0)
I	3	4 (44.5)	3 (15.8)
I	2	−	1 (5.3)
I	1	1 (11.1)	3 (15.8)
II central	3	1 (11.1)	2 (10.5)
II central	2	−	1 (5.3)
II	3	−	5 (26.3)
II	2	−	−

**Table 3:** Clinical characteristics of patients with and without retinal detachment.

Characteristic	Patients with retinal detachment (N = 9)	Patients without retinal detachment (N = 19)	*P-value*
Gestational age (weeks) median and range	25 (23–28)	25 (23–30)	>0.1^a^
Birth weight (gram) median and range	766 (480–1400)	802 (520–1450)	>0.1^a^
APROP (N, %)	3 (33%)	4 (21%)	0.48338^b^
Zone I ROP (N, %)	8 (89%)	11 (58%)	0.10099^b^
BPD (N, %)	9 (100%)	17 (89%)	0.31246^b^
IVH (N, %)	9 (100%)	18 (95%)	0.48338^b^
NEC (N, %)	4 (44%)	10 (53%)	0.68573^b^
PDA Lig (N, %)	6 (67%)	8 (42%)	0.22477^b^
PVL (N, %)	1 (11%)	1 (5%)	0.57469^b^
No visual function	4 (44%)	1 (5%)	nkml

^a^Kolmogorov–Smirnov test; ^b^Chi-square test

Abbreviations: BPD, bronchopulmonary dysplasia; IVH, intraventricular haemorrhage; NEC, necrotizing enterocolitis; PDA Lig., patent ductus arteriosus ligation; PVL, periventricular leukomalacia.

### Visual responses

Visual responses were obtained in 23 of 28 (82%) treated children, also in 5 out of 6 children with unilateral retinal detachment. Of the 5 children not responding to visual stimuli, bilateral retinal detachment occurred in 3 of them. Two children had optic nerve atrophy (one with periventricular leukomalacia in medical history; the second child was diagnosed with West syndrome, agenesis of the corpus callosum and cerebellar hypoplasia).

## Discussion

The Gynaecological and Obstetric Hospital of University of Medical Sciences in Poznań, where the NICU is located, is the largest obstetric hospital in Poland and third biggest in Europe. Two percent of Poles, i.e. about 7000 children are born there each year. The total number of children born in the study period was 29,758. Therefore, the results of this study might provide an indication of the epidemiological situation of ROP treatment in Poland. All patients with severe forms of ROP were granted continuation of medical care after discharge from the hospital. Only two patients from the whole group of laser therapy plus ranibizumab injection failed to follow-up in the outpatient clinic. Three patients died. Therefore, our study group comprised of almost all patients that underwent combined treatment in our centre. The study group consisted of extremely premature children with various comorbidities who were given intravitreal injections of ranibizumab as salvage treatment. However, a large percentage of children whose treatment failed indicates its insufficient effectiveness.

Several reports have shown that, for the treatment of ROP in zone I, better results are obtained using simultaneous combined intravitreal bevacizumab injection and zone I sparing laser ablation^10^. Another option is laser ablation in two sessions^8^. Some studies are using anti-VEGF first and then repeat or move to laser photocoagulation if unsuccessful initially^13^. The anti-VEGF monotherapy seems an attractive alternative to laser treatment for APROP. Several groups have reported positive effects of first-line bevacizumab therapy for APROP, with favourable anatomical outcomes and no retinal detachment^2,5,16^. Anti-VEGF as first line treatment has additional benefits such as continuation of retinal vascularization so that less laser in the case of ROP progression or reoccurrence is needed, it also causes less myopia compared to laser photocoagulation.

Chen *et al*.^17^ compared anti-VEGF – bevacizumab and ranibizumab – monotherapy and showed similar efficacy in the regression of ROP. Wang *et al*.^9^ compared both drugs and demonstrated that ROP reactivation is more likely after ranibizumab injection. However, ranibizumab has a shorter half-life^18^ and a higher binding affinity to VEGF than bevacizumab, theoretically making it a better option for treatment in preterm infants when treatment effectiveness and systemic absorption or side effects are concerned. In the case of combined therapy, when laser ablation damages the blood-retinal barrier, the choice of ranibizumab seems to be particularly beneficial and justified. We use ranibizumab in our centre due to its potentially lower systemic toxicity, which in our opinion may reduce the risk of systemic complications in premature infants. The decision was made with the support of the neonatology team and hospital administration.

As far as results of our study are concerned comparison with other studies is difficult because of the variability of inclusion criteria, indications and outcome measures. Law *et al*. and Nazari used bevacizumab to improve visualisation^19,20^. Araz-Ersan *et al*.^11^ administered ranibizumab on average 13 days after laser treatment to decrease vascular activity. Retinal detachment occurred in 22% of eyes, which constitutes a similar result as that of our group. Other research showing the results of combined therapy comprises mostly of case studies^12,21,22^.

Our results demonstrate that retinal detachment was not observed in any of the six children (10 eyes) who had at least partial remission before reoccurrence of ROP. In this group of patients, one child was diagnosed with APROP, three children with ROP in zone I with plus disease and two children with ROP in the central zone II with plus disease. Retinal detachment occurred only in the eyes with ROP progression or no apparent remission after laser therapy. The average time between laser therapy and ranibizumab injection was shorter in this group, on average 15 days compared to 31 days in children with primary ROP regression. Probably a simultaneous laser and anti-VEGF treatment or significantly shorter time between laser therapy and injection would improve the results.

In the same centre between September 2005 and October 2006, a prospective study included a group of 100 children born ≤30 weeks of gestation. Thirty children (60 eyes) were treated with laser photocoagulation. In 24 of them (80%), complete remission was observed and in six (12 eyes) further progression of the disease (20%) was diagnosed. In all cases with progression, it was zone I ROP and in all cases bilateral retinal detachment occurred. During this period, anti-VEGF had not yet been used^23^.

In the present study, similar results were observed as far as regression (83%) and progression (17%) rates after laser photocoagulation are concerned. However, in the group of laser treatment failure (63 eyes), regression of the disease with additional therapy with anti-VEGF was obtained in 65% of eyes (41 eyes). This result would not be possible to achieve without anti-VEGF therapy. This means a significant improvement in treatment results in comparison with previous research in our centre.

Since the corrected age of children from the study group varied from 7.5 months to 5 years, it was not possible to assess the visual responses using one method. The obtained functional results are difficult to compare with the studies of other authors. Kim^10^, in a group of 18 eyes treated with simultaneous anti-VEGF injection and laser therapy, and Mota *et al*.^12^ in a paper describing two cases treated with combination therapy evaluated only anatomical outcomes. Araz-Ersan *et al*.^11^ analysed anatomical outcomes and neurodevelopmental disabilities in 13 patients treated with simultaneous laser photocoagulation and bevacizumab injection. Wu *et al*.^13^, in 14 eyes after injection with further laser therapy, assessed the anatomical state and refraction in the treated eyes. In our study, despite the most advanced forms of ROP and the need for a two-stage treatment, visual function was preserved in the majority of children (82.1%).

Analysing the comorbidities of 28 enrolled children, it needs to be taken into consideration that this group was comprised of seriously ill premature babies who usually develop most severe stages of ROP. The comparison of this group of children with the group of children treated with laser therapy only would be inadequate, as the latter were usually in a better general condition. Also, the relationship between comorbidities and anti-VEGF therapy remains uncertain. This treatment remains controversial due to unknown long-term systemic safety. Hoerster *et al*.^24^ studied serum VEGF levels in infants treated with ranibizumab. They demonstrated a systemic reduction in VEGF levels, with readings below laboratory detection limits for 2 to 3 weeks, but eventually returning to normal at 4 weeks post-injection. Hong *et al*.^25^ showed that intravitreal bevacizumab injections significantly reduced plasma VEGF concentration in infants with ROP over a 7-week period. VEGF is critical for growth and development of vital organs such as kidneys, lungs and brain during the third trimester of pregnancy. Young age, possibly due to lower weight and an impaired blood-retinal barrier, increases anti-VEGF serum concentrations^3^. We are aware of the fact that anti-VEGF injections should be considered with great caution for ROP treatment. Some authors have expressed concern about the impact on neurodevelopment subsequent to anti-VEGF administration in premature babies^4^. Further studies are needed to investigate whether ranibizumab has less influence than bevacizumab on serum VEGF levels in ROP patients. Long-term studies should also be carried out to assess the usefulness anti-VEGF for zone II disease.

Based on our own experience and literature data^16^, we have now changed our treatment protocol so that APROP and zone I ROP are initially treated with intravitreal ranibizumab, while zone II retinopathy is still treated with laser ablation. The two-stage treatment of laser photocoagulation and ranibizumab injection might be used in the case of late referrals as they usually have advanced stages of ROP and are at high risk of retinal detachment. In these patients simultaneous laser and injection might be most beneficial.

The limitations of our study include data from a single institution, their retrospective character and the relatively small sample size. However, due to the lack of data from Central and Eastern European populations, we believe it is important to report the outcomes of this study.

A future study involving more centres would be beneficial to examine the effectiveness of the used treatment protocols in the search for the best treatment options in ROP patients.

## Conclusions

Ranibizumab injection can be taken into consideration in the selected cases of laser therapy failure. Children who do not achieve full remission after first-line therapy should be under special care as they are at high risk of developing retinal detachment. As the results of the two-stage procedure performed in our medical centre analysed in this study were unsatisfactory in comparison to the results of other authors, we have introduced another treatment regimen for the most severe ROP stages.
